# Stress Keratin 17 Is a Predictive Biomarker Inversely Associated with Response to Immune Check-Point Blockade in Head and Neck Squamous Cell Carcinomas and Beyond

**DOI:** 10.3390/cancers15194905

**Published:** 2023-10-09

**Authors:** Taja Lozar, Israa Laklouk, Athena E. Golfinos, Niki Gavrielatou, Jin Xu, Christopher Flynn, Aysenur Keske, Menggang Yu, Justine Y. Bruce, Wei Wang, Cvetka Grasic Kuhar, Howard H. Bailey, Paul M. Harari, Huy Q. Dinh, David L. Rimm, Rong Hu, Paul F. Lambert, Megan B. Fitzpatrick

**Affiliations:** 1 McArdle Laboratory for Cancer Research, University of Wisconsin School of Medicine and Public Health, 6459 Wisconsin Institute for Medical Research, 1111 Highland Ave., Madison, WI 53705, USA; tlozar@wisc.edu (T.L.); wwang93@wisc.edu (W.W.);; 2 University of Wisconsin Carbone Cancer Center, Madison, 53705 WI, USA; 3 University of Ljubljana, 1000 Ljubljana, Slovenia; cgrasic@onko-i.si; 4 Department of Pathology and Laboratory Medicine, University of Wisconsin School of Medicine and Public Health, MC 8550, 600 Highland Ave, Madison, WI 53792, USAcflynn4@wisc.edu (C.F.);; 5 Department of Pathology, Yale University, New Haven, CT 06510, USA; 6 Department of Biostatistics and Medical Informatics, University of Wisconsin School of Medicine and Public Health, Madison, WI 53705, USA; 7 Department of Medicine, University of Wisconsin School of Medicine and Public Health, Madison, WI 53705, USA; 8 Institute of Oncology Ljubljana, 1000 Ljubljana, Slovenia; 9 Department of Human Oncology, University of Wisconsin School of Medicine and Public Health, Madison, WI 53705, USA

**Keywords:** stress keratin 17, cytokeratin 17, predictive biomarker, immune check-point inhibitors, pembrolizumab, head and neck cancer, spatial transcriptomics, biomarker validation

## Abstract

**Simple Summary:**

Between 15 and 35% of head and neck cancer patients respond to immune check-point blockade therapy (ICB), and some may experience potentially life-threatening adverse events. Biomarkers that can reliably predict response to therapy are needed to improve patient selection. Our pilot data suggest the expression of cytokeratin 17 in pretreatment tumor samples predicts response to ICB in head and neck cancer. The aim of this study was to interrogate two independent patient cohorts to validate these observations and develop a robust CK17 immunohistochemical assay. Our study revealed cytokeratin 17 may be an independent predictive biomarker of inferior response to ICB in head and neck cancer. In an ICB-treated cohort of 552 patients with various cancer types, cytokeratin 17 RNA expression was predictive of patient survival.

**Abstract:**

Low response rates in immune check-point blockade (ICB)-treated head and neck squamous cell carcinoma (HNSCC) drive a critical need for robust, clinically validated predictive biomarkers. Our group previously showed that stress keratin 17 (CK17) suppresses macrophage-mediated CXCL9/CXCL10 chemokine signaling involved in attracting activated CD8+ T cells into tumors, correlating with decreased response rate to pembrolizumab-based therapy in a pilot cohort of ICB-treated HNSCC (*n* = 26). Here, we performed an expanded analysis of the predictive value of CK17 in ICB-treated HNSCC according to the REMARK criteria and investigated the gene expression profiles associated with high CK17 expression. Pretreatment samples from pembrolizumab-treated HNSCC patients were stained via immunohistochemistry using a CK17 monoclonal antibody (*n* = 48) and subjected to spatial transcriptomic profiling (*n* = 8). Our findings were validated in an independent retrospective cohort (*n* = 22). CK17 RNA expression in pembrolizumab-treated patients with various cancer types was investigated for predictive significance. Of the 48 patients (60% male, median age of 61.5 years), 21 (44%) were CK17 high, and 27 (56%) were CK17 low. A total of 17 patients (35%, 77% CK17 low) had disease control, while 31 patients (65%, 45% CK17 low) had progressive disease. High CK17 expression was associated with a lack of disease control (*p* = 0.037), shorter time to treatment failure (*p* = 0.025), and progression-free survival (PFS, *p* = 0.004), but not overall survival (OS, *p* = 0.06). A high CK17 expression was associated with lack of disease control in an independent validation cohort (*p* = 0.011). PD-L1 expression did not correlate with CK17 expression or clinical outcome. CK17 RNA expression was predictive of PFS and OS in 552 pembrolizumab-treated cancer patients. Our findings indicate that high CK17 expression may predict resistance to ICB in HNSCC patients and beyond.

## 1. Introduction

Immune check-point blockade (ICB)-mediated therapy is rapidly emerging as a valuable approach for treating cancer patients; however, response to ICB differs among patients and cancer types, including head and neck squamous cell carcinoma (HNSCC) [1]. Two programmed cell death protein 1 (PD-1) inhibitors, nivolumab and pembrolizumab, are approved for the treatment of patients with recurrent or metastatic HNSCC in the second line (platinum-refractory disease) as well as pembrolizumab alone or in combination with platinum and 5-fluorouracil in the first-line treatment of selected patients. However, only 13–18% of platinum-resistant patients [2] and 19–36% of HNSCC patients receiving pembrolizumab in the first-line setting respond favorably (i.e., complete or partial response) even when the tumor expresses high PD-L1 (PD-L1 combined positive score (CPS) ≥1 and ≥20) [3], suggesting only a small fraction of HNSCC patients benefit from ICB. There is a critical need for robust biomarkers that more reliably predict response to ICB and could thereby improve treatment selection in this patient population.

Our group previously showed that the expression of stress keratin 17 (also known as cytokeratin 17, CK17) alters the immune landscape in an HNSCC mouse model, contributing to resistance to ICB [4]. CK17 is a type I intermediate filament protein that is expressed during embriogenesis but silenced in mature somatic tissues, except in certain stem cell populations [5,6] and epithelial appendages [7]. CK17 expression can be induced in response to tissue injury, viral infections [8], psoriasis [9], and cancer [10,11]. Furthermore, high CK17 protein expression has previously been identified as a prognostic marker in several cancer types, including HNSCC [12,13,14,15,16,17,18,19]. 

The precise mechanisms of CK17 related to cancer prognosis is not known. CK17 is involved in multiple carcinogenesis pathways, such as transcription regulation and subcellular localization, glycolysis, enhancing cancer stemness, and others [20]. Recent data suggest that CK17 may be contributing to changes in tumor T cell infiltration, a critical mechanism of immune escape [21]. Studies in a mouse papillomavirus infection model and syngeneic HNSCC mouse model have shown that inducing CK17 expression suppresses T cell-mediated immune surveillance, resulting in an inverse correlation between CK17 expression and CD8+ T cell infiltration, regardless of HPV infection status [4,22]. Furthermore, experiments using an HNSCC mouse model demonstrated that knocking-out CK17 leads to reduced tumor growth rate, an influx of infiltrating CD8+ T cells, and increased responsiveness to ICB [4]. Our pilot analysis of 26 HNSCC patients treated with pembrolizumab-based therapy suggests that CK17 status, as determined via immunohistochemistry (IHC), correlates with lack of disease control with ICB and a shorter progression-free survival (PFS) and overall survival (OS) [4], generating the hypothesis that CK17 expression may be an informative biomarker for predicting inferior response to ICB in HNSCC patients. In the present study, we examined the expression of CK17 in an expanded discovery cohort as well as an independent validation cohort of HNSCC patients treated with ICB to further investigate the clinical relevance of CK17 in ICB-treated HNSCC and provide a comprehensive analysis of the predictive value of CK17 and its association with PD-L1 and other clinicopathologic characteristics in accordance with the Reporting Recommendations for Tumor Marker Prognostic Studies (REMARK) guidelines [23]. In addition, we used spatial transcriptomics to investigate the differential gene expression signatures in the tumors of responders and non-responders to pembrolizumab-based therapy. Lastly, we investigated a prognostic dataset of pembrolizumab-treated patients of various cancer types to assess the potential for translation beyond HNSCC.

## 2. Methods

### 2.1. Patient Selection

#### 2.1.1. Discovery Set (UW Cohort)

Patients with sufficient archival HNSCC tissue and medical record data that received at least 1 cycle of pembrolizumab-based therapy as part of routine clinical management at the University of Wisconsin-Madison between 1 January 2017 and 1 July 2022 were included in the discovery set (UW cohort). The last available sample prior to initiation of ICB therapy was selected for analysis. When available, additional samples per patient were obtained to assess concordance in staining patterns between primary and metastatic sites and pre- and post-chemoradiation. Demographic, clinical, radiographical, and treatment data for each patient were obtained from a retrospective chart review. Of the 53 eligible patients initially identified, 5 were excluded due to insufficient tissue. Finally, 48 patients were included in the study (Supplemental Figure S1).

#### 2.1.2. Validation Set (Yale Cohort)

The validation cohort (Yale cohort, YTMA523) comprised retrospectively collected pretreatment biopsy samples from 22 HNSCC cases treated with pembrolizumab at Yale’s New Haven Hospital from 2014 to 2020. After hematoxylin and an eosin-slide assessment by a trained pathologist, selected representative tumor areas were included in two independent tissue microarray (TMA) paraffin blocks, each block containing one non-adjacent 0.6 mm tumor-tissue core per case. ICB therapy outcomes were collected for all cases, including an assessment of the best documented response as defined with Response Evaluation Criteria in Solid Tumors (RECIST) version 1.1 and PFS.

### 2.2. Immunohistochemistry 

Archival formalin-fixed, paraffin-embedded (FFPE) tissue samples were stained for CK17 (Anti-Cytokeratin 17, Rabbit Monoclonal, clone EP1623, dilution 1:100, ab109725, Abcam, Cambridge, United Kingdom), PD-L1 (clone 22C3 for the discovery cohort and clone E1L33N for the validation cohort, respectively), and p16 (E6H4). Both a rabbit monoclonal and polyclonal CK17 antibody (ab109725 and ab53707, respectively) underwent extensive validation using CK17 highly expressing human SCC tissue and CK17 negative tonsil tissue as well as CK17 wild-type and knock-out mouse tissue (as previously described in [22]). Ultimately, clone ab109725 was selected based on the highest specificity for CK17. HPV status was determined based on p16 status for oropharyngeal tumors [24] and high-risk HPV RNA in situ hybridization for non-oropharyngeal tumors [25]. Detailed protocols are available in Supplemental Data S1.

### 2.3. Sample Evaluation and Development of a CK17 IHC Scoring System

Semi-quantitative evaluation of CK17 expression levels using brightfield microscopy was performed by two surgical pathologists (MBF and IL), one was blinded to the study endpoints. The staining intensity (weak (1+), moderate (2+), or strong (3+)) and percentage of tumor cells with any cytoplasmic staining were scored. Strong intensity corresponded with that in control samples used as standard. Non-invasive precursor lesions, immune cells, nuclear staining, necrotic cells, and keratin debris were excluded from the evaluation. A cutoff was determined using receiver operating characteristic analysis for disease control rate (DCR), the highest interobserver agreement based on an independent assessment by four blinded board-certified pathologists (JX, IL (review performed after a 6-month wash-out period), CF, and RH, Supplemental Table S1), and the highest intraobserver agreement (MBF, Supplemental Table S1). The independent blinded pathologists were instructed to score the percentage of strong (3+) positive stained tumor cells. Cases with strong (3+) cytoplasmic staining intensity in ≥25% of invasive tumor cells were grouped as high expressors. Cases with strong staining in <25% of tumor cells, or any percentage of low or moderate cytoplasmic staining intensity of tumor cell staining, were grouped as low expressors. The TMA slide was reviewed for the presence of an evaluable tumor; cores lacking an evaluable tumor or with fewer than 100 cells were excluded. 

### 2.4. PD-L1 and p16 IHC Interpretation

PD-L1 expression was re-scored for all patients according to CPS [26]. PD-L1 and p16 were scored by an experienced anatomic pathologist (MBF).

### 2.5. Next-Generation Sequencing

DNA extracted from FFPE tissue samples for 37 HNSCC patients from the UW cohort was analyzed using two next-generation sequencing (NGS)-based assays: Foundation Medicine (FoundationOne, Foundation Medicine, Cambridge, MA, USA, n = 2) or StrataNGS (test version 3, Strata Oncology, Ann Arbor, MI, USA, n = 35), ordered by clinicians as part of routine clinical practice. Both methods for the NGS-based clinical cancer gene assays used have been previously published, and assay performance has been rigorously validated [27,28].

### 2.6. Spatial Transcriptomics

Spatial transcriptomic profiling was performed on 51 selected regions of interest (ROIs) from 10 HNSCC archival pretreatment tissue samples (FFPE) from 10 selected patients included in the discovery cohort (UW-Madison) using the Nanostring Geomx Digital Spatial Profiler (DSP) using the Cancer Transcriptome Atlas gene panel. Patient samples were selected based on tissue availability and response status (3 responders and 7 non-responders to ICB). All experiments were performed in the Translational Initiatives in Pathology (TRIP) Laboratory at UW-Madison. Slides were incubated overnight with the Cancer Transcriptome Atlas (CTA) RNA probes in a HybEZ (ACD, Lansing, MI) oven at 37° with a HybriSlip^TM^ (Grace Bio-Labs, Bend, OR, USA) coverslip. The following day, the coverslip was removed, and slides were incubated for 1 h at room temperature with Nanostring’s Solid Tumor Morphology Marker Kit for staining CD45, PanCK, and DNA (SYTO 13) along with a custom marker, Anti-Cytokeratin 17 (1:100) conjugated to Alexa Fluor^®^ 647 (Ab196199, Boston, MA, USA). Geometric (circular) and custom regions of interest (ROIs) were selected based on visualization markers to generate tumor (PanCK+ and CK17+) and tumor immune microenvironment (TIME, PanCK−, CK17−, CD45+) areas. Slides were loaded onto the GeoMx Digital Spatial Profiler Instrument (Nanostring, Seattle, WA, USA) where the slide was scanned, and ROIs were selected on the image. ROIs were then segmented into PanCK-expressed regions (tumor compartment) vs. non-PanCK-expressed regions (non-tumor compartment). Following probe hybridization, UV cleavage, and barcode collection, gene expression was quantitated using PCR amplification. RNA Libraries were sequenced on a NovaSeq 6000. Paired-end, 150 bp sequencing was performed. Data were processed with bcl2fastq. For the RNA DSP assay, seven additional ROIs were missing, and four did not pass sequencing quality control. These cores were excluded from further analyses. Data analysis was performed using the NanoStringNCTools (version 1.5.0) and GeomxTools (version 3.1.1) Bioconductor packages in the R framework. We primarily used default segment quality control (QC) criteria: minSegmentReads = 1000, percentTrimmed = 80, percentStitched = 80, percentAligned = 75, percentSaturation = 50, inNegativeCount = 1, maxNTCCount = 9000, minNuclei = 20, and minArea = 1000. Out of 173 sequenced segments, 129 passed QC. The limit of quantification (LOQ) threshold was set at one standard deviation above the negative probe. All other parameters were set according to recommendations from the GeomxTools vignette. Differentially expressed genes were calculated with a Wilcoxon rank-sum test using counts per million normalized data with a Benjamini–Hochberg FDR correction and were plotted using version 3.4.0 of the ggplot2 R package. Representative marker genes for macrophages and T follicular helper cells were sourced from the available literature [29,30,31]. Hierarchical clustering of ROIs was performed and presented using the R package heatmap (version 1.0.12), showing representative marker genes and differentially expressed genes. Deconvolution of GeoMx ROIs was completed using CIBERSORT [32].

### 2.7. Pan-Cancer Analysis

Kaplan–Meier (KM) plotter (http://kmplot.com/analysis/, accessed on 24 September 2023), an open access platform for prognostic analysis, was used to assess the relationship between clinical outcomes and KRT17 expression in ICB-treated cancers [33]. Pretreatment samples from pembrolizumab-treated cancer patients in the “Immunotherapy” list were selected (n = 525 from Kovacs et al. [34]). We performed a prognostic analysis based on KRT17 expression levels in using this web-based tool. We calculated the hazard ratios (HRs), 95% confidence intervals (CIs), and log-rank *p* values. The median value was set as the cut-off. Sub-analyses according to cancer type could not be performed due to access limitations.

### 2.8. Study Endpoints

The primary endpoint was DCR, i.e., the percentage of patients with either radiographic response or stable disease as the best documented overall response to their ICB therapy. Response to ICB was investigator-assessed (TL) for all patients with at least one post-treatment scan or evidence of clinical progression after treatment initiation. Progressive disease included radiographic progression based on RECIST 1.1. [35] and/or clinical progression. Clinical progression was defined by deterioration of performance status leading to best supportive care/hospice or death in patients without restaging scans available at the time of analysis (n = 5/48). Secondary endpoints included PFS, time to treatment failure (TTF), OS, and differential gene expression between responders and non-responders. TTF was defined as the time in months from initiation of ICB to its discontinuation for any reason. PFS was defined as the time in months from initiation of ICB to the time of progression or death of disease, whichever comes first. OS was defined as the time in months from initiation of ICB until time of death from any cause.

### 2.9. Statistical Analysis

Statistical significance was investigated using the Chi-square test or Fisher’s exact and unpaired t-test for categorical and continuous variables, respectively, with an acceptable significance value of *p* < 0.05. TTF, PFS, and OS outcomes were estimated using the Kaplan–Meier method and log rank test. The Cox proportional hazard model was used for multivariate analysis. Variables considered for inclusion in the model were as follows: age at start of ICB, smoking status, tumor location, metastatic disease at start of ICB, keratinizing tumor, HPV status, and concurrent and prior chemotherapy and/or radiation. There were some variations in the timing and interval of radiological assessment, given the retrospective nature of this analysis. Median follow up was calculated for subjects without progression and was defined as median time from initiation of treatment to last known follow up with their provider. The statistical significance of the percentage of strong positive (3+) tumor cells between positive and negative cases was determined using the independent samples’ t-test. Interobserver variability data were analyzed using the Fleiss’ Kappa test. 

## 3. Results

### 3.1. Discovery Set

A total of 48 patients diagnosed with HNSCC were included in the discovery analysis (Table 1). Median age at ICB initiation was 64.0 years, 29 (29/48, 60%) were male, and 39 (39/48, 81.8%) had ECOG performance status ≤1. The most frequent anatomic location of the tumor was the oropharynx (21/48, 43.8%), followed by oral cavity (16/48, 33.3%). All 48 patients received pembrolizumab; of those, 39 (39/48, 81.3%) received pembrolizumab alone, 5 (5/48, 10.4%) in combination with 5-fluorouracil/carboplatin, and 4 (4/48, 8.3%) in combination with a phase I/II study agent (1 patient SNS-301 and 3 patients NKTR-214). A total of 17 patients (17/48, 35.4%) had disease control, while 31 patients (31/48, 64.5%) had progressive disease as best documented response to ICB. There were no significant differences in patient or tumor characteristics between patients having disease control and those with progressive disease (see Table 1). At the time of data cut-off, 41 (41/48, 85.4%) patients had disease progression, 34 (34/48, 70.8%) had died, 4 (4/48, 8.3%) discontinued ICB due to adverse events, and 3 (3/48, 6.3%, all achieving disease control) are still on treatment. Median follow-up was 17.5 months (95%, 9.84–32.36).

### 3.2. CK17 Expression in Pembrolizumab-Treated HNSCC

Whole slide sections were examined for CK17 immunohistochemical staining in the discovery cohort. Some tumors showed strong, diffuse staining. In cases with intratumoral heterogeneity, the average strong (3+) cytoplasmic expression was assessed and recorded. Nuclear expression and anything less than the strong cytoplasmic expression was considered negative. The same scoring system was later applied to the validation TMA. Varying degrees of strong (3+) cytoplasmic staining were observed in 38 (38/48, 79.2%) cases, with remaining cases showing no strong cytoplasmic CK17 expression in the invasive tumor component. The mean and median percentage of strong CK17 positive tumor cells in the invasive carcinoma component were 36.4% (SD 42.4) and 12.5% (IQR 90), respectively. Based on a cut-off of ≥25% of tumor cells with strong cytoplasmic staining, 21 (21/48, 43.8%) patients had CK17 high-expressing tumors, and 27 (27/48, 56.2%) had CK17 low-expressing tumors. In the CK17 high group, the mean and median percentage of strong (3+) CK17 positive tumor cells were 73.8% (SD 38.3) and 100% (IQR 65) vs. 7.2% (SD 10.7) and 1% (IQR 10.0) in the CK17 low group, *p* < 0.001. 

**Figure 1:**
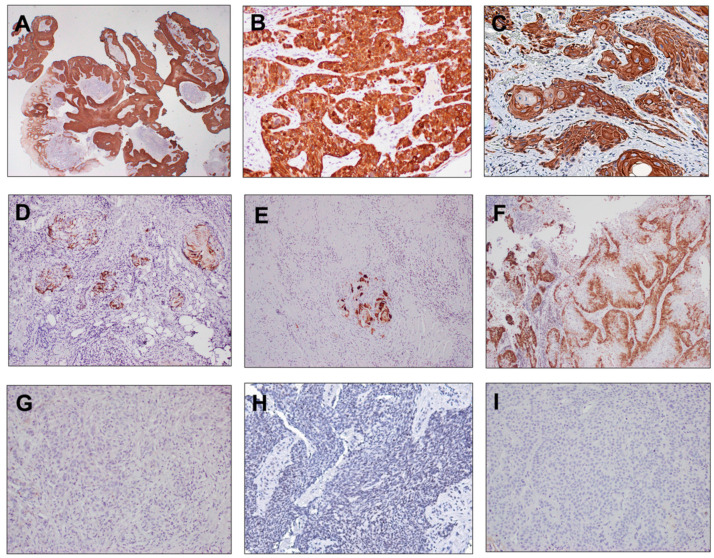
Stress keratin 17 (CK17) expression in head and neck squamous cell carcinomas treated with immune check-point blockade therapy. The expression patterns of stress keratin 17 (CK17) via immunohistochemistry and associated scoring are presented in representative cases. (**A**–**C**) (upper panel): high expression of CK17 with >25% tumor cells exhibiting strong (3+) cytoplasmic staining in the invasive tumor component (scored) and adjacent in situ component in (**A**) (not scored). (**D**–**F**) (middle panel): staining patterns considered ‘CK17 low’—(**D**,**E**): CK17 staining in <25% of tumor; (**F**): moderate intensity staining in a reserve cell/basal cell pattern of staining, <25% strong overall. (**G**–**I**) (lower panel): no expression of CK17. Magnification: (**A**) = 2× and (**B**–**I**) = 10×.

In the CK17 high group, uniform, strong (3+) cytoplasmic staining was observed in tumor cells of the invasive carcinoma component (Figure 1A–C), while the in situ component was not scored. The CK17 low group consisted of cases with <25% strong (3+) staining (Figure 1D,E) or cases with distinct staining patterns such as checkerboard-like patterns and reserve cell/basal cell patterns (17/27, 63%, Figure 1F) as well as cases with no expression of CK17 (10/27, 37%, Figure 1G–I). The expression of CK17 was consistent between primary tumors and matched distant metastasis (Supplemental Figure S2) and tended to better correlate with treatment outcome when the last available pretreatment sample before ICB was used (Supplemental Figure S3).

CK17 status was associated with histologic subtype such that CK17 high-expressing tumors were mostly keratinizing (17/21, 80.9%), and CK17 low-expressing tumors were mostly non-keratinizing (17/27, 63.0%), *p* = 0.003 (Table 2). When cases were analyzed separately via keratinization, CK17 did not predict DCR in non-keratinizing (*p* = 0.09) or keratinizing tumors (*p* = 1.0). No other association between CK17 status and other tumor characteristics was observed (Table 2). There was no correlation between CK17 status and HPV status or CK17 and PD-L1 status regardless of CPS cut-off (≥1%, ≥20%). One patient (1/48, 2.1%) had PD-L1 CPS<1 and had a CK17 high-expressing tumor. There were no statistically significant differences in patient and treatment characteristics between CK17 high and low groups (ECOG PS, extent of disease at ICB initiation, pembrolizumab regimen; *p* > 0.05, data not shown).

**Figure 2:**
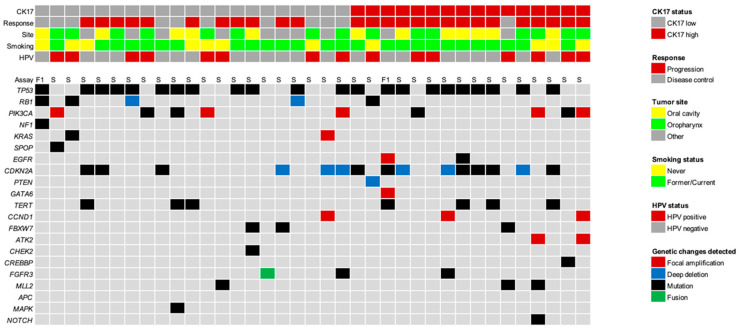
Illustration of somatic events as determined via next-generation sequencing (NGS) of clinically relevant cancer-related genes in the discovery cohort. No apparent correlation with CK17 expression can be observed. Relevant patient and tumor characteristics are presented in the top panel, above the genomic alterations. The color coding is explained in the key on the right. HPV—human papillomavirus, CK17—stress keratin 17, F1—Foundation One, and S—StrataNGS.

Furthermore, no association between CK17 expression and specific molecular alterations was observed among 37/48 (77.1%) patients with available NGS data (Figure 2). All patients were low in tumor mutation burden (TMB) and microsatellite stable.

### 3.3. Stress Keratin 17 Expression Predicts Lack of Disease Control from Pembrolizumab

**Figure 3:**
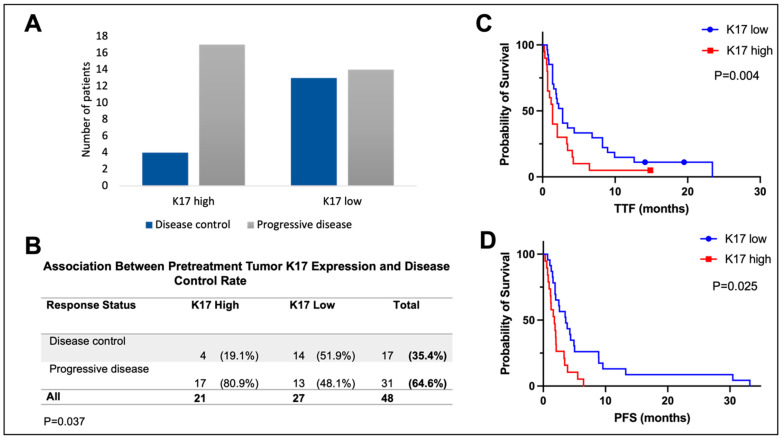
Stress keratin (CK17) status correlates with disease control rate, time to treatment failure (TTF), and progression-free survival (PFS) in immune-check-point-blockade-treated head and neck squamous cell carcinoma. (**A**,**B**): The correlation of CK17 immunohistochemistry (IHC) expression and disease control rate (**A**). CK17 expression via IHC was significantly correlated with lack of disease control (**B**). (**C**,**D**): Kaplan–Meier survival analysis. CK17 expression was significantly associated with TTF (panel **C**) and PFS (panel **D**).

DCR was associated with low CK17 expression status (*p* = 0.037, Figure 3A,B). In the CK17 high group, 17 (17/21, 81%) patients had progressive disease. In the CK17 low group, 14 (14/28 patients, 52%) had disease control, and 13 (13/28, 48%) patients had progressive disease.

CK17 expression was significantly associated with both TTF and PFS (Figure 3C,D). Median TTF was 1.38 months in the CK17 high group (95% CI 0.91–1.84) vs. 2.75 months in the CK17 low group (95% CI 1.81–3.70), *p* = 0.025. Median PFS was 1.78 months in the CK17 high group (95% CI 0.85–2.69) vs. 3.61 months in the CK17 low group (95% CI 1.65–5.57), *p* = 0.004. A similar trend was observed in the OS analysis (median OS 4.03 months (95% CI 1.72–6.34) vs. 7.34 (95% CI 4.93–9.83), *p* = 0.06, Supplemental Figure S4A). In the univariate Cox regression analysis, PFS was significantly associated with high CK17 expression (CK17 high vs. low: HR 2.58, 95% CI 1.32–5.08, *p* = 0.006, Supplemental Table S2) and keratinizing histology (HR 1.89, 95% CI 1.01–3.62, *p* = 0.049). In the multivariable Cox regression analysis, the only independent prognostic factor for PFS was CK17 (CK17 low vs. high: adjusted HR 2.21, 95% CI 1.08–4.54, *p* = 0.031). All tested variables are shown in Supplemental Table S2. 

### 3.4. Stress Keratin 17 Predicts Lack of Disease Control from Pembrolizumab in an Independent Validation Set

To validate our findings, we investigated an additional cohort of 22 patients with HNSCC treated with pembrolizumab-based therapy (median age of 63.0 years, 21/22 (95.5%) male, 17/22 (77.3%) former or current smokers, and 11/22 (50%) HPV-positive tumors), Table 3. There were 6/22 (27.3%) CK17 high- and 16/22 (72.7%) CK17 low-expressing tumors. There was no statistically significant correlation between DCR and clinicopathologic characteristics (Table 3) or between CK17 status and clinicopathologic characteristics (Supplemental Table S3). 

CK17 status was significantly correlated with lack of DCR (*p* = 0.011, Fisher’s exact) but not PFS (*p* = 0.174, not shown) or OS (*p* = 0.162, Supplemental Figure S4B). In the univariate Cox regression analysis, PFS was significantly correlated with concurrent chemotherapy (concurrent chemotherapy vs. no concurrent chemotherapy: HR 0.34, 95% CI 0.13–0.916, *p* = 0.033) and concurrent radiation (HR 2.05, 95%CI 1.07–3.93, *p* = 0.030). 

### 3.5. Spatial Transcriptomic Analysis Reveals Differentially Expressed Genes between Responders and Non-Responders

**Figure 4:**
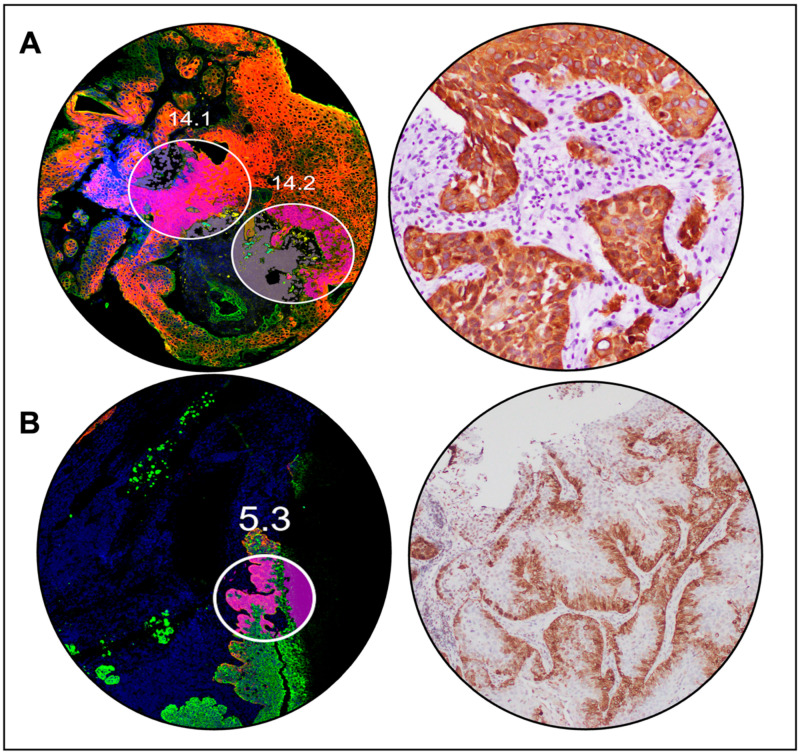
Representative cases of immune-check-point-blockade-treated head and neck squamous cell carcinomas with high vs. low stress keratin 17 (CK17) expression were selected for spatial transcriptomic profiling. (**A**): region of interest (ROI) selection (**left**) and H&E stain (**right**) on a representative case of CK17 high-expressing tumor from a patient with progressive disease on pembrolizumab. (**B**): ROI selection (**left**) and H&E stain (**right**) on a representative case of CK17 low-expressing tumor from a patient with disease control on pembrolizumab.

**Figure 5:**
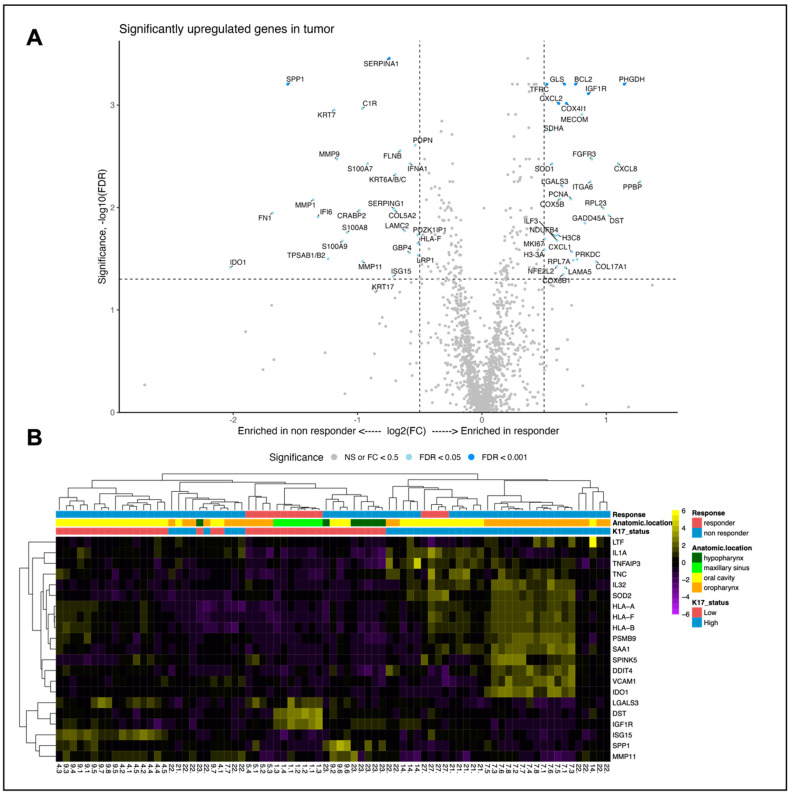
(**A**): differential gene expression in the tumor compartment (Pan-CK+) between non-responders and responders to pembrolizumab. (**B**): gene expression heatmap relative to key patient and tumor characteristics.

We selected 51 ROIs from 10 cases (three ICB responders and seven ICB non-responders, all pretreatment tissue) to investigate spatially resolved differential gene expression between tumors from responders and non-responders. Representative ROIs from CK17 high- (ROI 14.1 and 14.2) and CK17 low-expressing (ROI 5.3) tumors are shown in Figure 4A,B. Among the genes upregulated in the tumor compartment (79 PanCK+ segments from 10 patients) of non-responders (Figure 5B), the most differentially expressed were IDO1, HLA-F, S100A8, and S100A9. The expression of KRT17 was just below the level of significance. Three neutrophil chemokines, CXCL1, CXCL2, and CXCL8 were all found upregulated in the tumor compartment of responders (Figure 5B).

### 3.6. Pan-Cancer Analysis of KRT17 in Pembrolizumab-Treated Cancers

**Figure 6:**
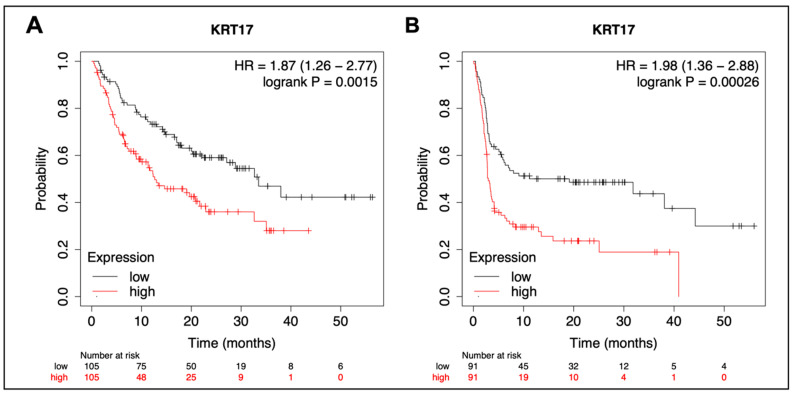
Exploratory pan-cancer analysis using publicly available transcriptomics data from pembrolizumab-treated patients. Kaplan–Meier curves were used to compare the overall survival (**A**) and progression-free survival (**B**) in K17 low- vs. high-expressing tumors.

A cohort previously published by Kovacs et al. [34] included 525 patients with solid tumors treated with pembrolizumab with available RNAseq from pretreatment tumor samples (40% responders). The cohort consisted of HNSCC, glioblastoma, melanoma, bladder, breast, gastric, and non-small cell lung carcinoma. Subset analysis by cancer type was unavailable for this dataset. KRT17 expression was significantly associated with OS (HR 1.87, 95% CI 1.26–2.77, *p* = 0.0015) and PFS (HR 1.98, 95% CI 1.36–2.88, *p* = 0.00026), Figure 6. ROC analysis revealed an AUC of 60% with a Mann–Whitney U test *p* value of 0.006 (Supplemental Figure S6).

## 4. Discussion

The present study aimed to evaluate the predictive value of CK17 in ICB-treated HNSCC according to the REMARK criteria as well as develop and validate a CK17 immunohistochemical assay. We demonstrate that CK17, as determined via IHC on whole tissue samples, is an independent predictive biomarker of the lack of response to ICB-based therapy in HNSCC. Furthermore, our findings suggest CK17 expression is independent of PD-L1 or HPV status. This is, to our knowledge, the first comprehensive evaluation of CK17 as a predictive biomarker of response to ICB in HNSCC. The present study adds to the growing body of literature on the role of CK17 as a diagnostic [36,37,38,39] and prognostic marker [13,40,41] in HNSCC. We further show the translational potential for CK17 as a predictive biomarker of ICB response in other cancer types.

While CK17 has been recognized as a negative predictive biomarker of resistance to gemcitabine in pancreatic carcinoma [19] and a positive predictive biomarker of response to ICB in colorectal carcinoma [42], our group is the first to have explored the role of CK17 as a biomarker of response to ICB in HNSCC [4]. To evaluate the strong association between CK17 status and lack of disease control from ICB therapy observed in our pilot study [4], the present study features a CK17 biomarker assay standardization (including an intraobserver and interobserver variability analysis) and biomarker assessment (in an expanded discovery and an additional validation cohort), and an analysis of the correlation between K17 and clinicopathologic characteristics, NGS mutation profile, effect size analysis, and a hypothesis-driven spatial transcriptomic experiment. The findings of the present study suggest the association between high CK17 expression and non-response to ICB is maintained in an expanded patient cohort as well as an independent validation set. Based on the discovery cohort, CK17 is an independent predictive marker of PFS when adjusted for variables identified in the univariate analysis (keratinizing histology); however, we did not show this in the validation cohort. The lack of association is likely related to the small sample size and smaller number of events as well as the higher percentage of patients treated with a combination of pembrolizumab and another agent compared to the discovery set (55% vs. 20%).

Interestingly, HPV status was not prognostic for PFS in our study. Published data suggest the independent prognostic value of HPV status is site-dependent [43], which may also be true for CK17. Due to the small sample size, sub-analyses on both HPV status and CK17 relative to anatomic location were not performed but should be considered in future studies.

In the present study, the percentage of CK17 high-expressing tumors was 41.6% in the UW cohort (discovery cohort and whole tissue) and 27.3% in the Yale cohort (validation cohort, TMA). We found a statistically significant association between DCR and CK17 in both the UW and Yale cohorts and a statistically significant association between PFS and CK17 in the UW, but not Yale cohort. This could be related to the differences between the study populations (31% in the Yale cohort vs. 10% in the UW cohort received chemotherapy with pembrolizumab). Also, the use of a single TMA core may be underestimating the extent of CK17 high expressors in the Yale cohort due to limited tissue, especially in cases with low tumor cell numbers per core.

The prognostic value of CK17 was first established by Regenbogen et al. [13] in a cohort of 78 oral, oropharyngeal, and laryngeal HNSCC of various disease stages where CK17 status was found to predict OS based on strong (2+) staining cut-off of 85% tumor cells as determined with PathSQ score across all anatomic locations. However, in their sub-analysis using a multivariate regression model, the respective optimal threshold differed by anatomic location, i.e., 85% for oral and 20% for oropharyngeal. We believe due to the differences in scoring methodology as well as disease stage and subsequent patient management of investigated cohorts, this threshold may not be directly compared to the present study. Furthermore, we have previously reported challenges related to scoring the heterogeneous staining pattern that fell beyond uniform tumor cell staining [4]. Regenbogen et al. described several CK17 staining patterns (such as checkerboard-like pattern and scattered positive cells), which are in line with our observations. According to our analysis, these patterns do not seem to hold any predictive value, and we believe CK17 should be grouped as low as previously described in a pilot dataset [4]. Here, we propose a scoring system to optimize translational potential while maintaining good interobserver and intraobserver agreement and high predictive value for both DCR and PFS. We evaluated CK17 staining intensity in whole slide tissue specimens, ranging from primary tumor tissues to distant metastasis and pre- vs. post- chemoradiation tissue specimens. The scoring system presented here-in takes into account all of the observed intra-tumoral and intra-patient heterogeneity. While our pilot data [4] were based on 5% strong positive tumor cell staining cut-off, the present analysis shows the cut-off of 25% strictly strong cytoplasmic staining offers improved interobserver variability while maintaining comparable predictive value.

The development of any biomarker immunohistochemical assay and scoring system has historically been challenging. The registration of pembrolizumab across various cancer types is associated with a specific PD-L1 diagnostic assay and an immunohistochemical score with specific cut-off values for patient selection [44]. For PD-L1 assessment, several staining platforms and scoring systems have been evaluated for reproducibility and interchangeability, and the effect of heterogeneous expression within a single tumor sample has been appreciated for some time [44,45]. No such data exist for CK17. To our knowledge, this is the first study to provide data on the reproducibility of a CK17 immunohistochemical score and cut-off values in HNSCC. Our discovery study cohort consisted of 31 biopsies and 17 resection specimens of HNSCC, which we believe represent the variability of CK17 expression in HNSCC in the real-world setting. Our data suggest good concordance in CK17 expression scoring between pathologists. Future directions should include a multi-center reproducibility and interobserver variability analysis including prior training of pathologists. Based on our sub-analysis on the effects of prior chemoradiation on CK17 expression, we hypothesize the last available pre-ICB sample should be used in future studies.

In looking at differential gene expression relative to the response to ICB, there was a trend toward CK17 upregulation in patients who did not receive clinical benefit from ICB compared to responders. Another stress keratin, KRT6, was also upregulated in the tumor compartment of non-responders, consistent with the co-expression of stress keratin families seen in our previously published mouse model [22]. Two other genes were upregulated in patients that had not received clinical benefit to ICB: S100A8 and S100A9. S100A8 and S100A9 are two calcium-binding proteins that can be expressed and secreted via neutrophils, monocytes, and keratinocytes in the context of chronic inflammation [46]. These genes have been reported as markers for poor prognosis in several cancer types, including melanoma undergoing ICB therapy [47,48]. This is the first report correlating the expression of these genes to response to ICB in the context of HNSCC. Whether the expression of S100A8/S100A9 is a result of chronic inflammation related to cancer or plays a causative role in response to ICB therapy and patient prognosis requires further investigation. In addition, we identified SPP1 (also known as OPN, osteopontin) among the upregulated genes of non-responders. SPP1 is an integrin-binding matricellular protein that has been found to be involved in many cellular processes such as cell signaling pathways, cell adhesion and migration, cell-mediated immunity, angiogenesis, and metastasis [49]. SPP1 is a known negative prognostic marker in HNSCC [50] and appears to be predominantly expressed in macrophages [51,52] and to a much smaller extent in tumor cells in HNSCC [52]. Bill et al. recently reported that the CXCL9/SPP1 ratio reflects macrophage polarity and can predict response to anti PD-1 therapy in HNSCC patients [52]. The data reported here-in are consistent with these observations. Furthermore, we have previously reported an inverse correlation between CK17 and CXCL9/10 expression in preclinical models [53]. The relationship between CK17 tumor expression and CXCL9/SPP1 expressing macrophages should be explored further.

As the correlation between ICB response and CK17, as determined with spatial transcriptomics analysis, was just below the level of significance, we further investigated a publicly available dataset of pembrolizumab-treated cancers. We found CK17 expression with bulk RNAseq was a significant predictor of inferior PFS and OS with an AUC of 60%. Unfortunately, the subset data analysis was not available for this dataset. The predictive value of CK17 in cancer types included in this dataset (most notably, melanoma, gastric, and bladder cancer) has not been explored to date. Considering the positive association between CK17 and response to ICB in colorectal cancer [42], but not in HNSCC, further investigation into these cancer types may be of interest.

The main limitations of this study are related to the retrospective nature of this study and a small study sample size. We noted some differences between the discovery and validation cohort, namely the percentage of patients with disease control and percentage of patients that received first-line ICB. In addition, the TMA (Yale cohort) included a single 0.6 mm diameter tissue core per case, which may not be fully representative of patient CK17 status. Several studies have investigated the concordance of various stains between whole tissue sections and TMA cores and have shown that two to three 0.6 mm cores per case adequately represent the tissue heterogeneity observed in whole tissue sections [53,54]. When rigorous validation steps are applied, a single core per case has been shown to produce high-quality results [53]. While our analysis of CK17 staining in TMA cores served an exploratory purpose to support further prospective validation studies, future studies using TMAs should attempt to include several tissue cores per case and validate the number of cores needed for an accurate representation of CK17 expression in SCC.

## 5. Conclusions

This study demonstrates that high CK17 protein expression is an independent predictive biomarker of the lack of disease control from pembrolizumab-based therapy in HNSCC. CK17 expression is independent of PD-L1 or HPV status. Future considerations include validating the scoring system of CK17 and its predictive value for ICB therapy in the prospective setting.

## Figures and Tables

**Table 1:** Patient characteristics, discovery cohort.

Characteristic, N (%)	All Patients N = 48		Disease Control N = 17		Progressive Disease N = 31		*p* Value
	N	%	N	%	N	%	
Age, median (years, IQR)	64.0	11.8	66.0	7.5	61.0	14.0	0.295
Sex							0.885
Female	9	18.8	3	17.6	6	19.4	
Male	29	60.4	14	82.4	25	80.6	
ECOG performance status ≥2	9	18.8	2	11.8	7	22.6	0.460
Current or former smoker	34	70.8	12	70.6	22	71.0	0.978
Primary tumor location							0.185
Oral cavity	16	33.3	3	17.6	13	41.9	
Oropharynx	21	43.8	9	52.9	12	38.7	
Larynx	2	4.2	1	5.9	1	3.2	
Nasopharynx	4	8.3	3	17.6	1	3.2	
Other *	5	10.4	1	5.9	4	12.9	
Prior systemic therapy	37	77.0	13	76.5	24	77.4	0.761
HPV-positive tumor	21	43.8	10	58.9	11	35.5	0.119
PD-L1 expression (CPS)							1.0
<1	1	2.1	0	0	1	3.2	
1–19	24	50.0	9	52.9	15	48.4	
≥20	23	47.9	8	47.1	15	48.4	
Front-line therapy							0.089
Surgery	22	45.8	4	23.5	18	58.1	
Chemoradiation	18	37.5	10	58.8	8	25.8	
Neoadjuvant Chemotherapy	1	2.1	0	0	1	3.2	
Palliative Chemotherapy	3	6.3	2	11.8	1	3.2	
Radiation only	4	8.3	1	5.9	3	9.7	
Single-agent pembrolizumab regimen	39	81.3	14	82.4	25	80.6	1.0
Concurrent radiation	8	16.7	3	17.6	5	16.1	1.0
Received ICB first line	19	39.6	4	23.5	15	48.3	0.127
Metastatic disease at initiation of ICB	36	75.0	13	76.5	23	74.2	0.862
Number of doses, mean (range)	3.0	(1–35)	11.6	(2–35)	2.9	(1–7)	<0.001
Median TTF, months (95% CI)	1.9	1.4–3.3	8.3	5.9–10.7	1.4	1.1–1.7	<0.001
Median PFS, months (95% CI)	2.0	1.3–3.4	6.5	0.1–13.0	1.9	1.5–2.3	<0.001
Median OS, months (95% CI)	6.1	2.1–10.1	8.3	5.9–10.8	4.6	1.1–8.2	0.168
Discontinuation due to AEs	4	8.3	3	17.6	1	3.2	0.084

CPS—combined positive score, ECOG—Eastern Cooperative Oncology Group, ICB—immune check-point blockade, IQR—interquartile range, HPV—human papillomavirus, PD-L1—programmed death ligand 1, AE—adverse events, TTF—time to treatment failure, and PFS—progression free survival, OS—overall survival. * Includes hypopharynx, paranasal sinuses, and unknown.

**Table 2:** Association between stress keratin 17 (CK17) protein expression and clinicopathologic parameters, discovery cohort.

Characteristic	All Cases N = 48		CK17 Protein Expression				
			CK17 High N = 21		CK17 Low N = 27		*p* Value
	N	%	N	%	N	%	
Tumor stage at diagnosis							0.683
T0	1	2.1	0	0	1	3.7	
T1	6	12.5	2	9.5	4	14.8	
T2	9	18.8	5	23.8	4	14.8	
T3	5	10.4	1	4.8	4	14.8	
T4	27	56.3	13	61.9	14	51.9	
Lymph node stage at diagnosis							0.149
0	7	14.6	3	14.3	4	14.8	
1	2	4.2	1	4.8	1	3.7	
2	25	52.1	14	66.7	11	40.7	
3	14	29.2	3	14.3	11	40.7	
Primary tumor location							0.274
oral cavity	16	33.3	8	38.1	8	29.6	
oropharynx	21	43.8	10	47.6	11	40.7	
larynx	2	4.2	1	4.8	1	3.7	
nasopharynx	4	8.3	1	4.8	3	11.1	
other *	5	10.4	0	0	5	18.5	
Histologic subtype **							0.003
keratinizing	27	56.3	17	80.9	10	37.0	
non-keratinizing	21	43.8	4	19.1	17	62.9	
Sample type							0.769
biopsy	31	64.6	13	61.9	18	66.7	
resection	17	35.4	8	38.1	9	33.3	
Tissue type							0.462
primary tumor	19	39.6	7	33.3	12	44.4	
local recurrence	16	33.3	10	47.6	6	22.2	
locoregional lymph node metastasis	5	10.4	2	9.5	3	11.1	
distant metastasis	8	16.7	6	28.6	2	7.4	
Received radiation before obtaining study tissue	24	50.0	10	47.6	14	51.9	1.0
HPV-positive tumor	21	43.8	10	47.6	11	40.7	0.771
PD-L1 expression (CPS)							NC
<1	1	2.1	1	4.8	0	0	
1–19	24	50.0	9	42.9	15	55.6	
≥20	23	47.9	11	52.4	12	44.4	
Received chemotherapy before obtaining study tissue	22	45.8	10	47.6	12	44.4	0.624
Received radiation before obtaining study tissue	24	50.0	10	47.6	14	51.9	1.0
Concurrent chemotherapy while on ICB	8	16.7	6	28.6	2	7.4	0.115
Concurrent radiation while on ICB	8	16.7	6	28.6	2	7.4	0.115

CPS—combined positive score, ICB—immune check-point blockade, HPV—human papillomavirus, PD-L1—programmed death ligand 1, and NC—not calculated. * Includes hypopharynx, paranasal sinuses, and unknown. ** Based on matched H&E stain at the time of CK17 assessment.

**Table 3:** Patient characteristics, validation cohort.

Characteristic	All Patients N = 22		Disease Control N = 14		Progressive Disease N = 8		*p* Value
	N	%	N	%	N	%	
Age, median (years, IQR)	63.1	13.6	63.1	16.4	61.2	14.9	1.0
Sex							1.0
Female	1	4.5	1	7.1	0	/	
Male	21	95.4	13	92.9	8	100.0	
Current or former smoker	17	77.3	11	78.6	6	75.0	1.0
Primary tumor location							NC
Oral cavity	2	9.1	1	7.1	1	12.5	
Oropharynx	12	54.5	7	50.0	5	62.5	
Larynx	3	13.6	2	14.3	1	12.5	
Other *	5	22.7	4	28.6	1	12.5	
HPV Status							NC
Positive	11	50.0	6	42.9	5	62.5	
Negative	9	40.1	6	42.9	3	37.5	
Missing	2	9.1	2	14.2	0	0	
PD-L1 expression (CPS)							0.086
<1	5	22.7	4	28.6	1	12.5	
1–19	9	40.9	3	21.4	6	75.0	
≥20	8	36.4	7	50.0	1	12.5	
Single-agent pembrolizumab regimen	9	40.1	4	28.6	5	62.5	0.187
Concurrent radiation	7	31.8	5	35.7	2	25.0	0.671
Received ICB first line	14	63.6	8	57.1	6	75.0	0.649
Metastatic disease at initiation of ICB	18	81.8	11	78.6	7	87.5	1.0
Median PFS, months (95% CI)	7.3	1.8–12.8	15.1	3.2–27.0	1.3	0.5–2.1	<0.001
Median OS, months (95% CI)	18.8	4.5–33.1	31.1	4.6–57.6	4.0	0.1–8.6	0.001

CPS—combined positive score, ICB—immune check-point blockade, IQR—interquartile range, HPV—human papillomavirus, NC—not calculated, PD-L1—programmed death ligand 1, and PFS—progression-free survival, OS—overall survival. * Includes hypopharynx, paranasal sinuses, and unknown.

## Data Availability

The data that support the findings of this study are available from the corresponding author (P.F.L. and M.B.F.) upon reasonable request.

## Supplementary Materials

The following supporting information can be downloaded at https://www.mdpi.com/article/10.3390/cancers15194905/s1, Data S1: Immunohistochemistry protocols; Figure S1: Study flow chart; Figure S2: Immunohistochemistry expression of K17 is maintained between primary tumor site and distant metastases and across metastatic sites; Figure S3: Expression of stress keratin 17 (K17) by immunohistochemistry in tissue samples prior and post chemoradiation; Figure S4: Overall survival (OS) analysis in the discovery and validation cohorts; Figure S5: Examples of distinct immune signatures based on computational deconvolution using CIBERSORT of the spatial transcriptomic data; Figure S6: Receiver operating characteristic analysis using ROC Plotter platform (https://www.rocplot.org/, accessed on 24 September 2023) on the Immunotherapy-treated cohort (pembrolizumab only, pretreatment, all tumor types); Figure S7: High-risk HPV RNA in-situ hybridization was performed on all non-oropharyngeal cases with sufficient tissue available. Table S1: Summary of the receiver operating characteristics (ROC) analysis and interobserver variability analysis; Table S2: Univariate and multivariate analysis of clinicopathologic variables with PFS, discovery cohort; Table S3: The association between stress keratin 17 (K17) protein expression and clinicopathologic parameters, validation cohort.
