# Supplementary material for: Stress Keratin 17 Is a Predictive Biomarker Inversely Associated with Response to Immune Check-Point Blockade in Head and Neck Squamous Cell Carcinomas and Beyond
Source: Cancers (Basel). 2023 Oct 9;15(19):4905. doi: 10.3390/cancers15194905 (PMC10571921; doi:10.3390/cancers15194905)

Supplemental Materials

Supplemental Figure 1. Study flow chart.

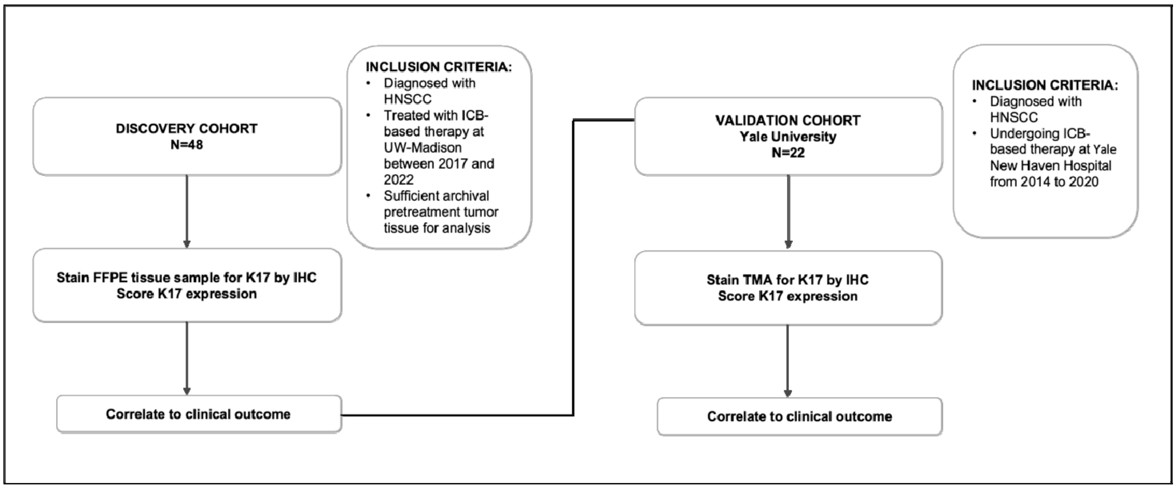

HNSCC - head and neck squamous cell carcinoma, FFPE - formalin fixed, paraffin embedded, ICB - immune check-point blockade, IHC - immunohistochemistry, K17 - stress keratin 17, TMA - tissue microarray, UW - University of Wisconsin.

Supplemental Data 1. Immunohistochemistry protocols

Archival formalin-fixed, paraffin-embedded (FFPE) tissue samples were obtained from the UW Department of Pathology and Laboratory Medicine and sectioned into 4-µm-thick sections and deparaffinized according to standard procedures before being processed for IHC staining. The tissue block containing sufficient (at least 100 invasive carcinoma cells) was selected based on review of all available H&E slides from each case. Deparaffinization was carried out on the instrument, as was heat-induced epitope retrieval in the form of “cell conditioning” with CC1 buffer (Ventana, #950-224), an EDTA based buffer pH 8.4, for 32 minutes at 95◦C. IHC for K17 (Anti-Cytokeratin 17, Rabbit Monoclonal, Clone EP1623, dilution 1:100, ab109725, Abcam, Cambridge, United Kingdom), PD-L1 (clone 22C3) and p16 (E6H4) was performed on an automated stainer (Ventana Discovery Ultra BioMarker Platform (Roche, USA)) following the manufacturer’s instructions. For K17 IHC, a positive (human squamous cell carcinoma) and negative (human tonsil tissue) control were included with each run. RNA in-situ hybridization (ISH) was performed on all available archival specimens of non-oropharyngeal tumors with sufficient tissue for additional analysis. ISH for HPV E6/E7 transcript was completed using RNAscope (2.5 HD Reagent Kit-Brown, 322300, Advanced Cell Diagnostics, Newark, CA, USA) with probes specific for 18 high-risk HPV genotypes (probe 312591) according to the

manufacturer’s instructions. A positive control was included with each run, while adjacent benign tissue served as negative control. Stains were interpreted by an exprienced surgical pathologist (RH).

Supplemental Table 1. Summary of the receiver operating characteristics (ROC) analysis and interobserver variability analysis. ROC analysis examined the prognostic performance of stress keratin (K17) by percent strong positive cells to predict lack of disease control to immune check-point blockade-based therapy. The area under the receiver-operating characteristic (ROC) curve (AUC), which plots percentage sensitivity against 1 minus percentage specificity, was used to evaluate the prognostic performance of the K17 IHC assay (Supplemental Table 2). The optimal cutoff of the percentage of strong positive (3+) tumor cells in the invasive component to define positive and negative cases was chosen to balance sensitivity and specificity. The candidate cut-offs were 5% [1] 25% and 95%. AUC (ROC) =61.1%.

K17 tumor expression cut-off ≥	Cases, N (%)	Sensitivity	Specificity	Correlation with DCR (Chi-Square), p value	Correlation with PFS (log rank), p value	Interobserver agreement*			Intraobserver agreement**		
						κ for positivity	Agreement [49]	p-value	κ for positivity	Agreement [49]	p-value
5% strong (3+) cells	28 (58.3)	0.645	0.529	0.393	0.181	0.72	good	<0.001	0.24	poor	0.028
25% strong (3+) cells	20 (41.7)	0.484	0.706	0.037	0.004	0.73	good	<0.001	0.71	good	<0.001
95% strong (3+) cells	14 (29.2)	0.290	0.882	0.049	<0.001	0.70	good	<0.001	1.00	excellent	<0.001

*four pathologists
**reference pathologist

Supplemental Figure 2. Immunohistochemistry expression of K17 is maintained between primary tumor site and distant metastases (cases A, B - K17 low) and across metastatic sites (case C - K17 high). Case A: left: Representative image (negative K17) of a biopsy of a local recurrence at the primary tumor site (neck mass). A-right: Biopsy of the right posterior ileum in the same patient (negative K17). Case B: left: Resection of the primary tumor of the pharynx (negative K17). B-right: Biopsy of a liver metastasis (negative K17). Case C: right: Biopsy, liver metastasis (positive K17). C-left: Biopsy, lung metastasis in the same patient (positive K17).

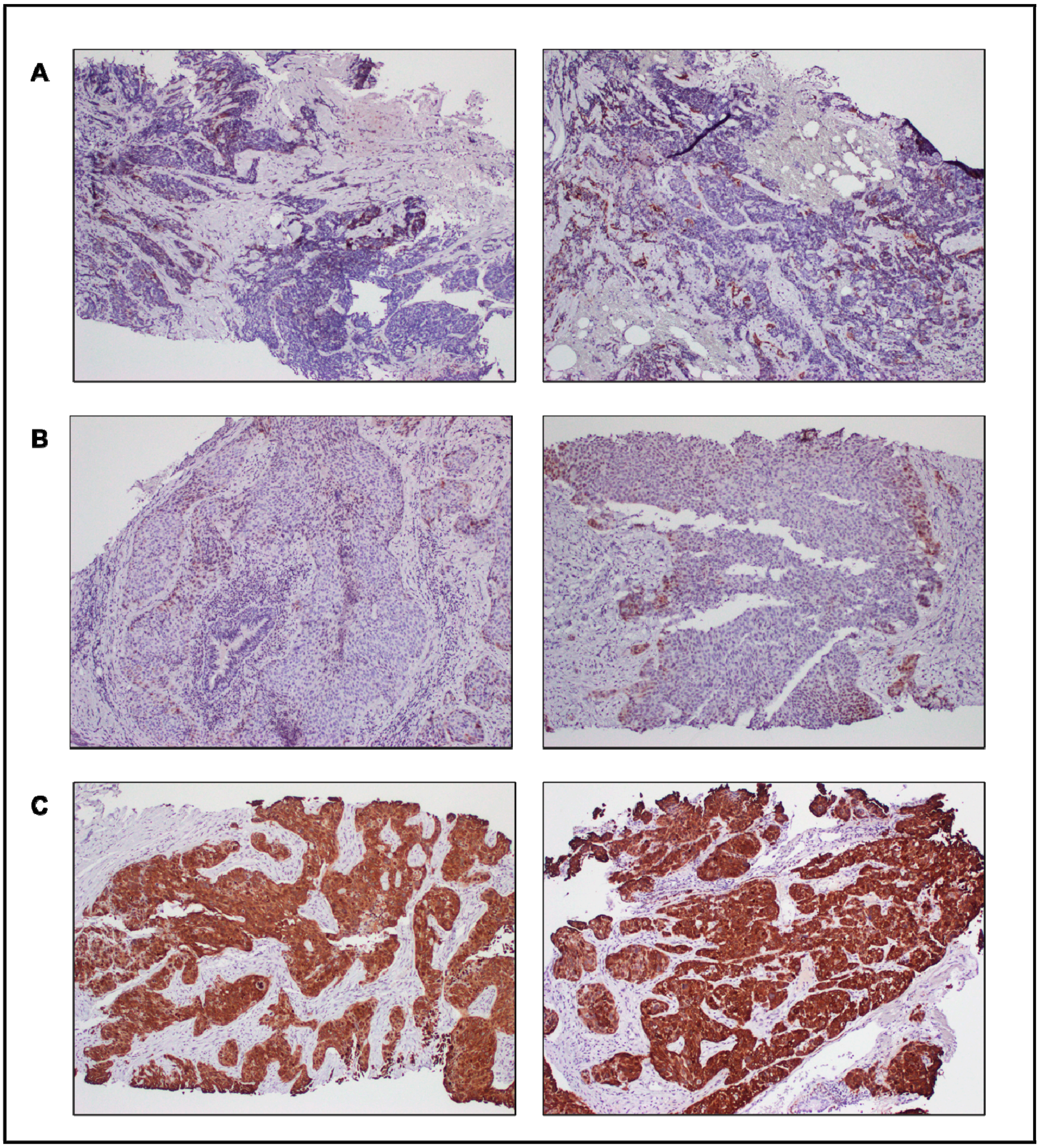

Supplemental Figure 3. Expression of stress keratin 17 (K17) by immunohistochemistry in tissue samples prior and post chemoradiation. Case A: K17 low, A-left: Representative image of a biopsy of the primary tumor (tongue) at diagnosis, no prior therapies. A-right: Biopsy of a local recurrence at primary tumor site, post chemoradiation. Case B: K17 high, B-left: Resection of the primary tumor (right tonsil), no prior therapies. B-right: Biopsy of a local recurrence at primary tumor site, post chemoradiation. Case C: discordant, patient with no disease control

from pembrolizumab. C-left: Resection of the primary tumor (right tonsil), considered K17 low. C-right: Biopsy of a liver metastasis, 7 years post chemoradiation, considered K17 high.

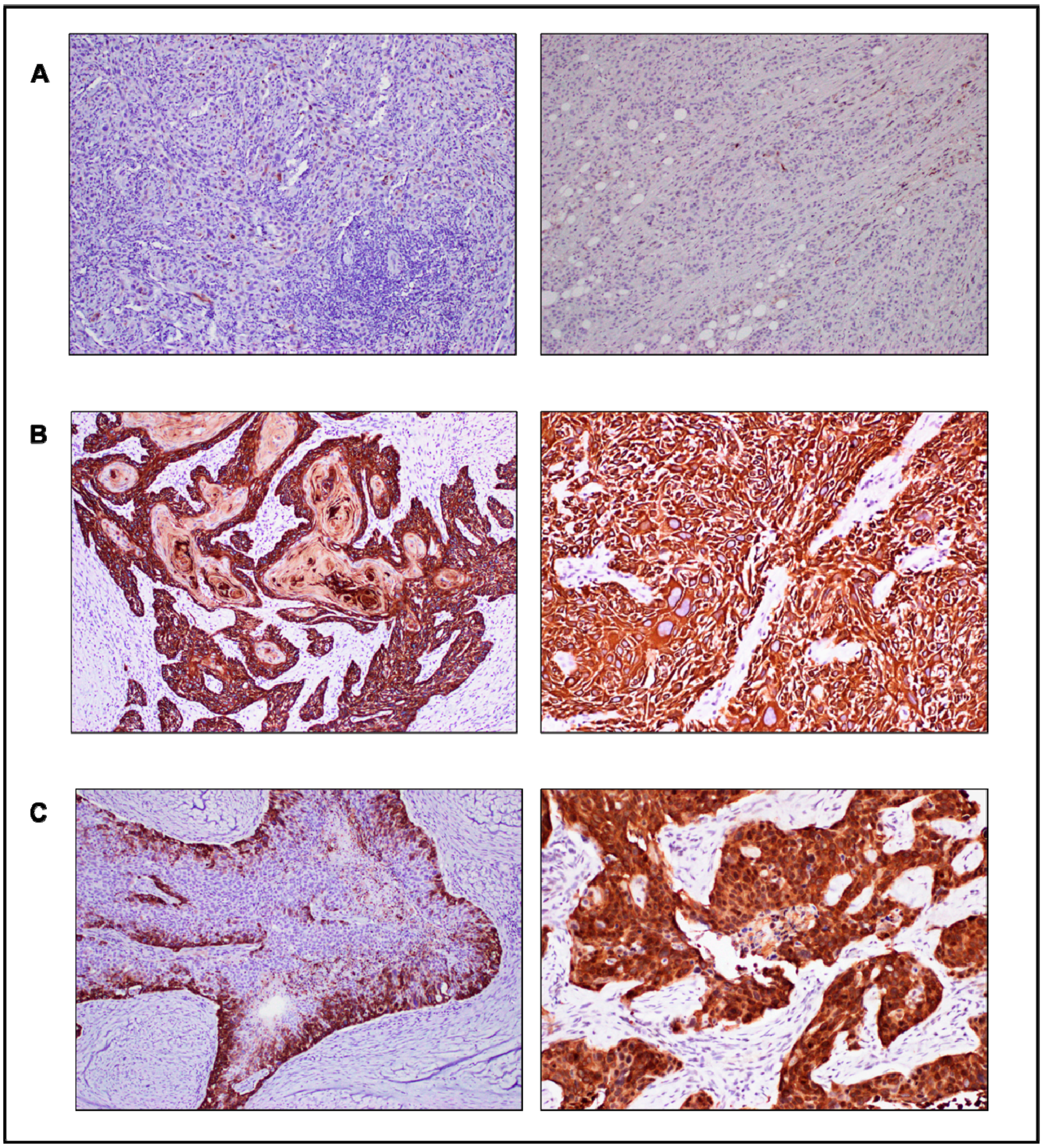

Supplemental Figure 4. Overall survival (OS) analysis in the discovery and validation cohorts. A. Discovery cohort. Median OS in the K17 high vs. low groups was 4.03 (95% CI 1.72-6.34) and 7.38 (95% CI 4.93-9.83), p=0.06. B. Validation cohort. Median OS in the K17 high vs. low groups was 5.3 (95%CI 0.86-9.74) and 31.1 (95% CI 13.91-48.3), p=0.162.

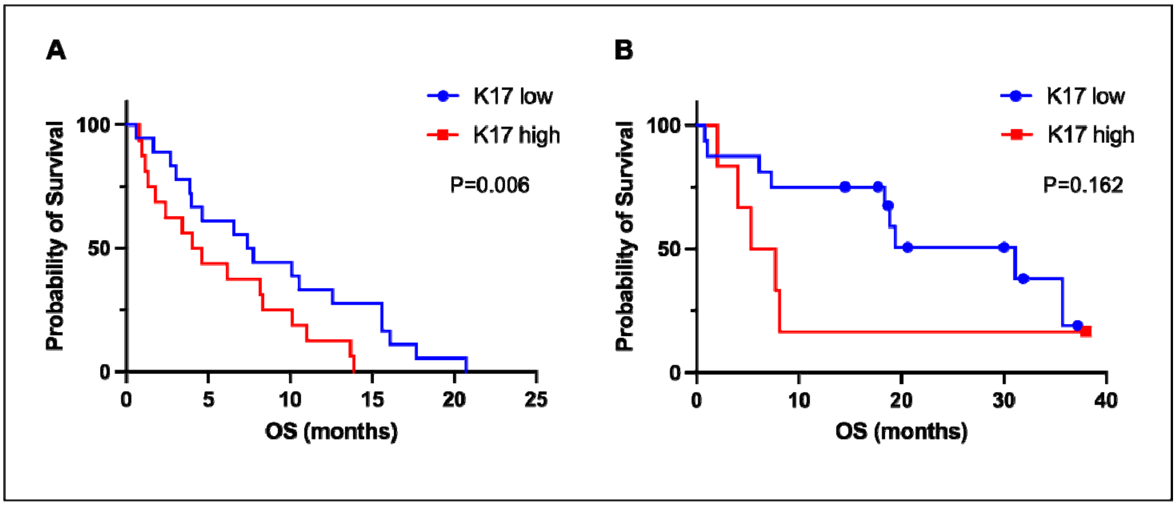

Supplemental Table 2. Univariate and multivariate analysis of clinicopathologic variables with PFS, discovery cohort. HR - hazard ratio, CI - confidence interval, ICB - immune-check-point blockade, HPV - human papillomavirus, PD-L1 - programmed death ligand 1, CPS - combined positive score.

**Reference variable**	**N (%) or median, years**	**Univariate analysis**			**Multivariate analysis**		
		HR	95%CI	p value	HR	95% CI	p value
Age at start of ICB	64.0	0.97	0.94-1.01	0.119			
K17 >25% strong staining	21 (43.8)	2.57	1.32-5.02	0.006	2.21	1.08-4.54	0.031
Distant metastasis at start of ICB	36 (75.0)	0.66	0.31-1.40	0.278			
Current/former smoker	34 (70.8)	1.11	0.58-2.13	0.759			
Oropharyngeal tumor	21 (43.8)	0.75	0.40-1.41	0.373			
HPV positive tumor	21 (43.8)	0.68	0.36-1.27	0.224			
Keratinizing histology	27 (56.3)	1.90	1.01-3.57	0.048	1.46	0.73-2.89	0.284
Concurrent chemotherapy	8 (16.7)	1.48	0.65-3.40	0.352			

Concurrent radiation	8 (16.7)	1.25	0.55-2.84	0.597		
Prior chemotherapy	37 (77.1)	0.67	0.30-1.50	0.329		
Prior radiation	42 (87.5)	1.26	0.38-4.18	0.701		
Platinum resistant disease*	13 (27.1)	0.72	0.35-1.49	0.372		
PD-L1 > 20 (CPS)	23 (47.9)	0.91	0.49-1.69	0.762		

* Platinum resistance was defined as progression <6 months following last platinum-based chemotherapy.

Supplemental Table 3. The association between stress keratin 17 (K17) protein expression and clinicopathologic parameters, validation cohort.

**Characteristic**	**All patients N = 22**		**K17 high N = 6**		**K17 low N= 16**		**p value**
	N	%	N	%	N	%	
**Age, median (years, IQR)**	63.1	13.6	63.7	14.9	61.8	17.7	**1.0**
**Sex**							**1.0**
Female	1	4.5	0	0	1	6.3	
Male	21	95.4	6	100.0	15	93.8	
**Current or former smoker**	17	77.3	6	100.0	11	68.8	**0.266**
**Primary tumor location**							**NC**
Oral cavity	2	9.1	0	/	2	12.5	
Oropharynx	12	54.5	5	83.3	7	43.8	
Larynx	3	13.6	1	16.7	2	12.5	
Other*	5	22.7	0	/	5	31.3	
**HPV Status**							**0.455**
Positive	11	50.0	3	50.0	8	50.0	
Negative	9	40.1	3	50.0	6	37.5	
Missing	2	9.1	0	/	2	12.5	
**PD-L1 expression (CPS)**							**0.482**
<1	5	22.7	1	16.7	4		
1-19	9	40.9	4	66.7	5		
≥20	8	36.4	1	16.7	7		
**Single agent pembrolizumab regimen**	9	40.1	3	50.0	6	37.5	**0.655**
**Concurrent radiation**	7	31.8	2		5	31.3	**1.0**
**Received ICB first-line**	14	63.6	4	66.7	10	62.5	**1.0**
**Metastatic disease at initiation of ICB**	18	81.8	5		13	81.3	**1.0**
**Median PFS months (95% CI)**	7.3	1.8-12.8	2.0	0.3-3.7	10.9	2.7-19.1	**0.174**

**Median OS months (95% CI)**	18.8	4.5-33.1	5.3	0.9-9.7	31.1	13.9-48.3	**0.162**

ICB - immune-check-point blockade, HPV - human papillomavirus, NC - not calculated. *Includes hypopharynx, paranasal sinuses, and unknown.

Supplemental Figure 5. Examples of distinct immune signatures based on computational deconvolution using CIBERSORT of the spatial transcriptomic data: T helper rich (top panel) and macrophage rich (bottom panel). Only 7 ROIs had sufficient detection rate in the CD45 compartment for downstream analysis.

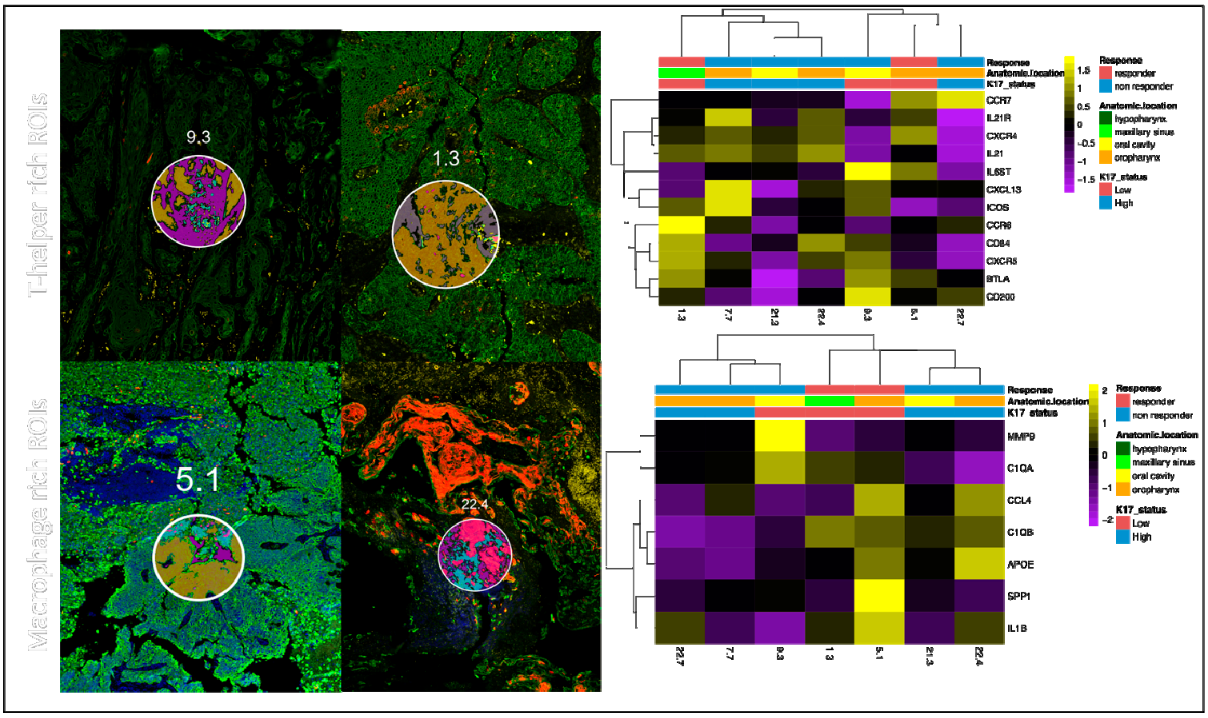

Supplemental Figure 6. Receiver operating characteristic analysis using ROC Plotter platform (https://www.rocplot.org/, accessed in 9/24/23) on the Immunotherapy-treated cohort (pembrolizumab only, pretreatment, all tumor types).

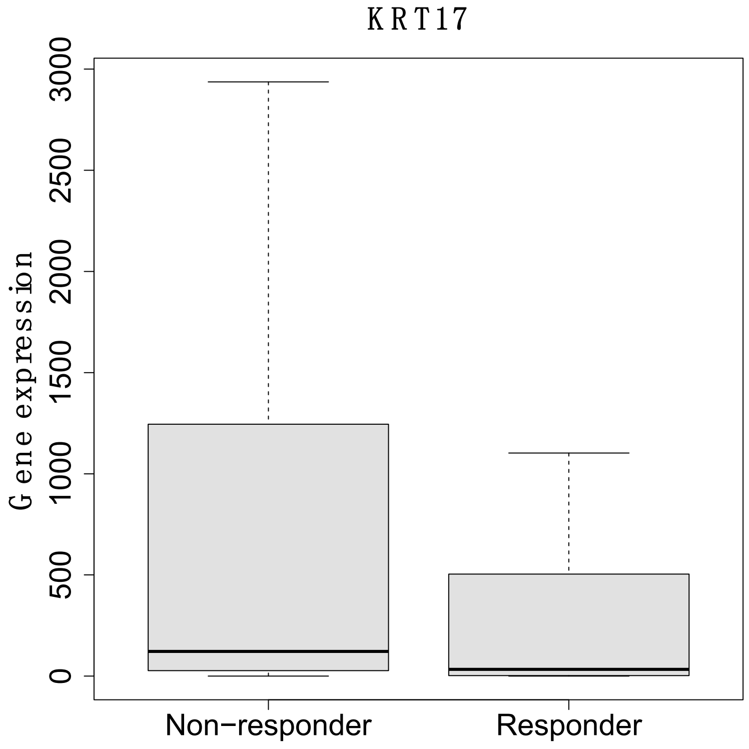
KRT17
Gene expression
0
500
1000
1500
2000
2500
3000
Non−responder
Responder

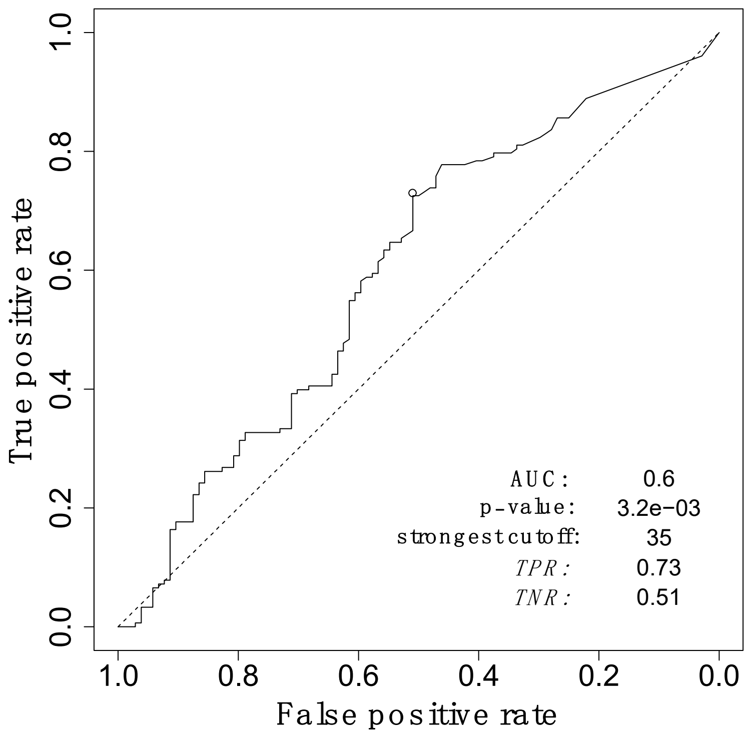
True positive rate
False positive rate
0.0
0.2
0.4
0.6
0.8
1.0
AUC: 0.6
p-value: 3.2e−03
strongest cutoff: 35
TPR: 0.73
TNR: 0.51

Supplemental Figure 7. High-risk HPV RNA in-situ hybridization was performed on all non-oropharyngeal cases with sufficient tissue available. A: Patient sample positive for HR-HPV. B: Positive control sample. C-D: Negative study patient samples.

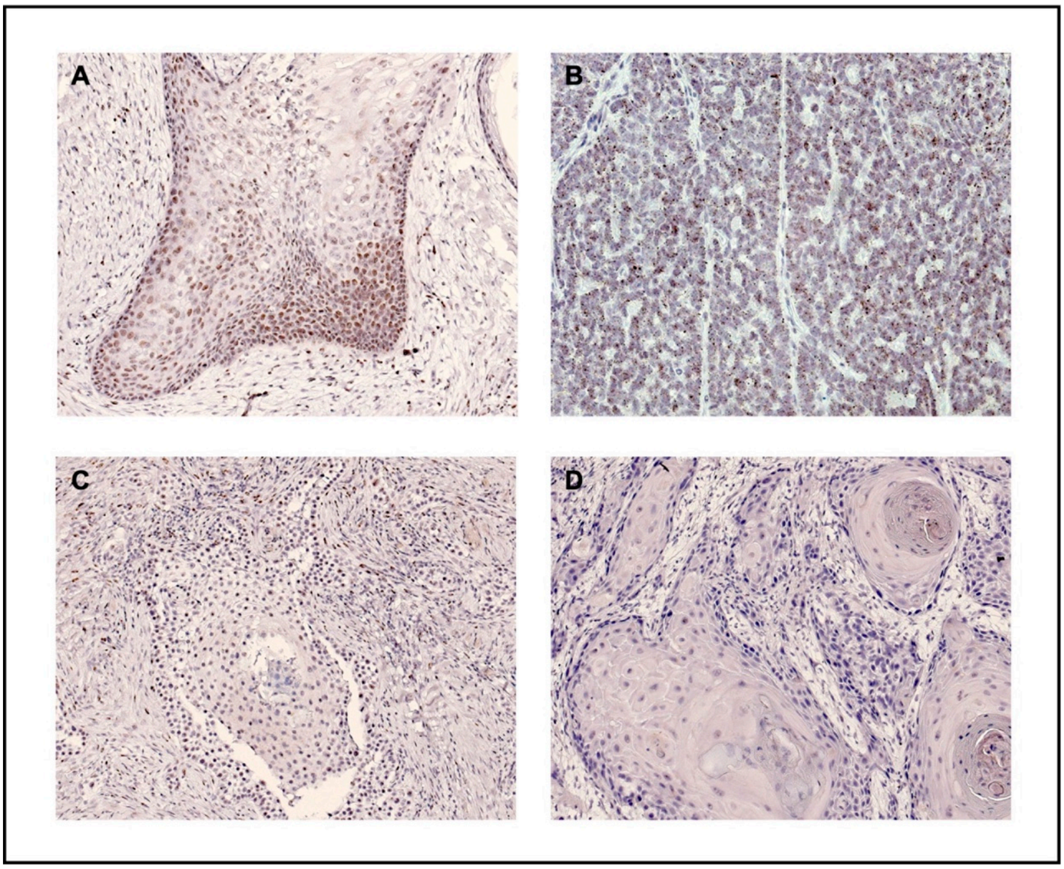

Supplement: Supplementary file 1 [file cancers-15-04905-s001.zip › cancers-2601578-supplementary.pdf]
